# Can relative muscle strength and relative hop performance cut-offs identify patients who sustain a second ACL injury?

**DOI:** 10.1016/j.jsampl.2026.100163

**Published:** 2026-11-05

**Authors:** Johan Högberg, Jakob Lindskog, Axel Sundberg, Ramana Piussi, Rebecca Hamrin Senorski, Roland Thomeé, Kristian Samuelsson, Eric Hamrin Senorski

**Affiliations:** a Sahlgrenska Sports Medicine Center, Gothenburg, Sweden; b Unit of Physiotherapy, Department of Health and Rehabilitation, Institute of Neuroscience and Physiology, Sahlgrenska Academy, University of Gothenburg, Box 455, SE-405 30 Gothenburg, Sweden; c Department of Orthopaedics, Institute of Clinical Sciences, Sahlgrenska Academy, University of Gothenburg, Gothenburg, Sweden; d Department of Orthopaedics, Sahlgrenska University Hospital, Mölndal, Sweden

**Keywords:** Anterior cruciate ligament, ACL, Muscle function, Return to sports, RTS, Knee injury

## Abstract

**Background:**

The aim was to determine cut-off values for relative isokinetic quadriceps and hamstring muscle strength and relative hop performance and evaluate their ability to discriminate between patients who sustained and did not sustain a second anterior cruciate ligament (ACL) injury within 2 years after returning to pre-injury level of sports following ACL reconstruction (ACLR).

**Methods:**

Data were extracted from a registry in August 2026. Patients aged 15–35 years at index ACLR using an ipsilateral autograft, rated ≥6 on the Tegner Activity Scale who returned to pre-injury level of sport, and had performed muscle strength and hop performance tests at time of return to pre-injury level of sport were included. Relative muscle strength was normalized to body mass and hop performance to body height. Receiver operating characteristic analyses were performed to determine cut-off values and discriminatory ability for second ACL injury within 2 years. Secondary analyses evaluated ipsilateral and contralateral ACL injuries separately.

**Results:**

428 patients were included (average age 21.5 ± 4.9 years; 50% female) of whom 50 (11.7%) sustained a second ACL injury within 2 years (31 ipsilateral and 19 contralateral). Relative muscle strength and hop performance demonstrated poor discriminatory ability for any second ACL injury, with AUCs ranging from 0.48 to 0.55. Discriminatory ability remained poor in side-specific analyses of ipsilateral (AUC 0.50–0.55) and contralateral (AUC 0.42–0.55) ACL injuries.

**Conclusion:**

Calculated cut-off values for relative strength and relative hop performance were unable to differentiate between patients who sustained and did not sustain a second ACL injury.

**Level of evidence:**

Prospective observational registry-study II.

**Clinical trial registration:**

Not applicable.

## 1 Introduction

Batteries of tests that consist of strength and hop performance assessments are recommended to evaluate knee function recovery after anterior cruciate ligament (ACL) reconstruction (ACLR) [1]. Patients are typically recommended to achieve ≥90% in relation to the uninjured leg (limb symmetry index [LSI]) in a battery of tests to inform decision-making prior to return to sports (RTS) [2]. However, 14–53% of patients fail to pass the cut-off value of ≥90% LSI in a battery of tests prior to RTS [3]. In cases where patients successfully pass the battery of tests, the current data does not support that passing is associated with reduced risk for second ACL injury [4], [5], [6].

The inability to use results from a battery of tests to discriminate between patients who sustain and do not sustain a second ACL injury may suggest that current batteries of tests, or the interpretation of their results, are not sensitive enough or appropriate to identify patients at risk for second ACL injury after RTS [4], [5], [6]. Simonsson et al. [6] reported that the use of LSI of muscle strength and hop performance tests, regardless of analyzed as cut-offs, incremental, or as a deviation from 100% (symmetry), could not differentiate between patients who sustained a second ACL injury from patients who did not. Although LSI can account for inter-individual variability, the muscle strength symmetry does not account for whether the muscle strength of the uninjured leg comparator for each patient decreases or increases [7], [8]. An alternative approach would be to calculate strength in relation to body mass and hop performance in relation to body height, which may provide more information of patients' muscle strength and knee function capacity than LSI. Body height may influence hop performance and may be used as a proxy for leg length when direct measures of leg length are unavailable [9]. Whether cut-off values based on relative strength and hop performance can discriminate between patients who sustain and do not sustain a second ACL injury after RTS has not yet been established. Furthermore, as second ACL injuries may occur either ipsilaterally or contralaterally, analysis of respective leg's muscle strength and hop performance may provide additional information.

The purpose of this study was to determine cut-off values for relative isokinetic quadriceps and hamstring muscle strength and relative hop performance and evaluate their ability to discriminate between patients who sustained and did not sustain a second anterior cruciate ligament (ACL) injury within 2 years after returning to pre-injury level of sports following ACLR. Secondary purposes were to evaluate ipsilateral and contralateral ACL injuries separately using relative muscle strength and hop performance of the corresponding leg.

## 2 Method

### 2.1 Study design

The present study was based on a rehabilitation specific registry, Project ACL, and reported according to the Standards for Reporting Diagnostic Accuracy (STARD) statement [10]. Ethical approval has been obtained from the Regional Ethical Review Board in Gothenburg (265–13, T023-17), and the Swedish Ethical Review Authority (2020–02501, and 2024-08724-01).

### 2.2 Project ACL: a rehabilitation registry

Project ACL was initiated 2014 in Gothenburg, Sweden, with the aim to improve the care for patients with ACL injuries regardless of age, activity level or choice of treatment. Upon registration, patients enter demographic information on Project ACL's website. Through a web-based protocol, patients are regularly invited (10 weeks, 4, 8, 12, 18, 24, 60 months, and then every fifth year) to complete patient-reported outcomes (PROs), and to perform isokinetic muscle strength tests and hop performance tests with ACL injury or ACLR as baseline. Participation is voluntary and informed consent is obtained during registration on Project ACL's website. Withdrawal can be done at any time.

### 2.3 Inclusion and exclusion criteria

Patients 15–35 years old at ACLR who had undergone an index ACLR with an autograft harvested from the ipsilateral side, rated ≥6 on the Tegner Activity Scale (Tegner) [11] pre-injury, returned to their pre-injury level of sport, and performed muscle strength and hop performance tests at the follow-up at which return to pre-injury level of sport was first reported were eligible for inclusion. Patients who did not sustain a second ACL injury and had less than 2 years of follow-up from returning to pre-injury level of sport were excluded, as were patients with a non-surgically treated contralateral ACL injury at the time of return to pre-injury level of sports.

### 2.4 Tegner Activity Scale

To assess the level of knee-strenuous activity, the Tegner was used. A modified Tegner is used in Project ACL [12], which rates sports and activities on a range from 1 to 10 in contrast to the original scale ranging from 0 to 10 [11]. Higher values indicate more knee-strenuous activities, where lower scores indicate less knee-strenuous activities. The Tegner has an acceptable test-retest reliability (intraclass coefficient (ICC) = 0.8) with acceptable floor and ceiling effects [13].

### 2.5 Battery of tests in project ACL

The battery of tests in Project ACL consists of isokinetic muscle strength assessment for the quadriceps and hamstrings as well as 3 hop performance tests: vertical hop, hop for distance, and the 30 s side hop test. All tests were supervised by physical therapists trained by Project ACL to ensure standardized administration of each test.

In accordance with the standardized Project ACL testing protocol, isokinetic muscle strength for the quadriceps and hamstrings was performed seated in a concentric mode in a dynamometer (Biodex System 4; Biodex Medical System, Shirley, NY, USA) using an angular velocity of 90° per second through a range of motion between 90° and 0° of knee flexion, with gravity correction applied [14]. A standardized test protocol was used which started with 10 min warm-up on a stationary bike, followed by ten practice trials in the dynamometer at 50% and 75% of perceived maximal effort, respectively. Patients were then asked to perform one repetition at 90% of maximal effort prior to the maximum attempts. After the warm-up procedure, the patients were asked to perform 3 to 4 single attempts with maximal effort, separated by 40 s of rest in between each attempt. The peak torque measured as Newton meter (Nm) was recorded and registered in the database.

All hop tests were performed with the patients holding their hands behind their backs. Patients were allowed 2 to 3 submaximal practice trials on each leg prior to the maximal attempts, starting with the ACLR leg. Assessment of hop tests started with the vertical hop test, for which patients were instructed to hop as high as possible with a straight knee and were permitted 3 maximal attempts for each leg. Flight time from take-off to landing was measured using MuscleLab (Muscle lab, Ergotest Technology, Oslo, Norway) and converted to jump height according to the equation $h=\frac{{\left(g*t\right)}^{2}}{8}$*,* where *h* is jumping height, *g* is gravitational acceleration (9.81 m/s^2^), and *t* is flight time. Jump height was expressed in centimeters (cm). Secondly, hop for distance was performed, for which patients were instructed to hop as far as possible while achieving a controlled landing and were permitted five maximum trials. For a successful trial, patients were required to perform a controlled landing i.e., landing on the jumping foot without releasing their hands from behind their backs, moving the jumping foot or supporting balance with the opposite foot. Distance in cm from toes at take-off to heel at landing was measured. Thirdly, the side hop test was performed past 2 lines 40 cm apart. Patients were instructed to hop as many times as possible for 30 s. In case of touching the lines, the repetition was counted as an error. The number of successful repetitions (total number of hops minus errors) were registered, as well as the total number of errors. No standardized verbal encouragement beyond the test instructions was specified in the Project ACL testing protocol. The hop tests described have been reported to have high reliability for test-retest and ability to discriminate between ACLR patients and healthy individuals [15].

### 2.6 Data collection

Data was extracted from Project ACL database in August 2026. Data for the respective tests were extracted from the first follow-up at which patients reported a return to their pre-injury level of sport and performed the muscle function tests. Return to pre-injury sport level was defined as reporting the same or higher Tegner rating compared with the pre-injury level. Thus, the follow-up at which return to pre-injury level of sport was first reported, rather than the actual date of return to pre-injury sport level, constituted the index time point for the present study.

Peak quadriceps and hamstring torque (Nm) were normalized to body mass (kg) to represent the muscle strength available relative to the body mass that the patient is required to control during weight-bearing sporting activities (relative muscle strength). Vertical hop and hop for distance were divided by body height (cm) and expressed as percentages. For the side hop test, the number of successful hops was divided by body height and expressed as a percentage, consistent with a previously reported approach [16]. Although the side hop outcome is a repetition count rather than a distance, each successful repetition requires the patient to traverse a fixed lateral distance of 40 cm. Body height was therefore used as an anthropometric normalization factor, despite that this normalization differs conceptually from normalizing hop height or distance. Anthropometric characteristics, including body height and leg length, may influence hop performance [9], [16]. As leg length was unavailable in the registry, body height was used as a surrogate measure. The included tests were analyzed as a battery of tests, as well as individual tests.

Patients or the responsible physical therapist can add information of an eventual second ACL injury (including both subsequent ipsi- and contralateral ACL injury) on the Project ACL website. Second ACL injuries were subsequently confirmed through clinical assessment and magnetic resonance imaging. Patients were followed for second ACL injury for 2 years from the index follow-up at which return to pre-injury sport level was first reported, or until sustaining a second ACL injury. Patients classified as not sustaining a second ACL injury were required to have at least 2 years of follow-up after the index follow-up. Second ACL injuries occurring more than 2 years after the index follow-up were not considered events for the present outcome. The 2-year follow-up was chosen due to second ACL injuries occur most frequently within 2 years of returning to sports [17], [18].

### 2.7 Outcome

The primary outcome was a second ACL injury. More specifically, the present study aimed to investigate the ability of relative quadriceps and hamstring muscle strength and relative hop performance to discriminate between patients who sustained and did not sustain a second ACL injury within 2 years of return to pre-injury sport level. Secondary analyses evaluated ipsilateral and contralateral ACL injuries separately. For ipsilateral second ACL injury, muscle strength and hop performance of the ACLR leg were evaluated, whereas for contralateral ACL injury, corresponding measures of the uninjured leg were evaluated.

### 2.8 Statistical analysis

Statistical analyses were performed using Statistical Product and Service Solutions (IBM Corp. Released 2017. IBM SPSS Statistics for Windows, Version 29.0. Armonk, NY: IBM Corp.). Patient demographics were presented as count with distribution for categorical variables and mean with standard deviations or median with the 25th and 75th percentiles for continuous variables. For comparisons, Fisher's exact test was used for categorical variables and independent t-test or Mann–Whitney U-test for continuous variables. An alpha level of 0.05 was set. To calculate cut-off values for relative muscle strength and hop performance for patients who sustained and did not sustain a second ACL injury, the receiver operating characteristics (ROC) curve was used. The ROC curve displays the discriminatory ability of a value to separate 2 outcomes, in this case, a second ACL injury or no second ACL injury. Specificity (true negatives), and sensitivity (true positives) were calculated, as well as the area under the ROC curve (AUC) to evaluate the accuracy of the calculated cut-off value to distinguish between patients who sustained and did not sustain a second ACL injury.

Receiver operating characteristic analyses were performed to evaluate the discriminatory ability of relative muscle strength and hop performance for second ACL injury within 2 years with second ACL injury defined as the positive state. Separate ROC analyses were performed for [1] any second ACL injury [2], ipsilateral second ACL injury, and [3] contralateral ACL injury. For the analysis of any second ACL injury and ipsilateral second ACL injury, test values from the ACLR leg were used. For contralateral ACL injury, test values from the uninjured leg were used. Patients who sustained a contralateral ACL injury were excluded from the ipsilateral analysis, and patients who sustained an ipsilateral ACL injury were excluded from the contralateral analysis. For each test, the AUC with 95% confidence interval (CI) was calculated to quantify discriminatory ability. Cut-off values were determined using the Youden's index (Sensitivity + Specificity – 1), with the maximum Youden's index used to identify the optimal cut-off. The Youden's index ranges from 0 to 1, where 0 indicates no discriminatory ability and 1 represents perfect discrimination. Sensitivity and specificity were reported for each cut-off. The following AUC reference values were used: <0.5 = no discriminative ability, ≥0.5 to <0.7 = poor, ≥0.7 to <0.8 = acceptable, ≥0.8 to <0.9 = excellent and ≥0.9 = outstanding discriminative ability [19].

## 3 Results

In August 2026, 5002 patients were registered in Project ACL. After application of inclusion criteria, 541 were eligible, and 428 patients were included after application of the exclusion criteria (Fig. 1).

**Fig. 1:**
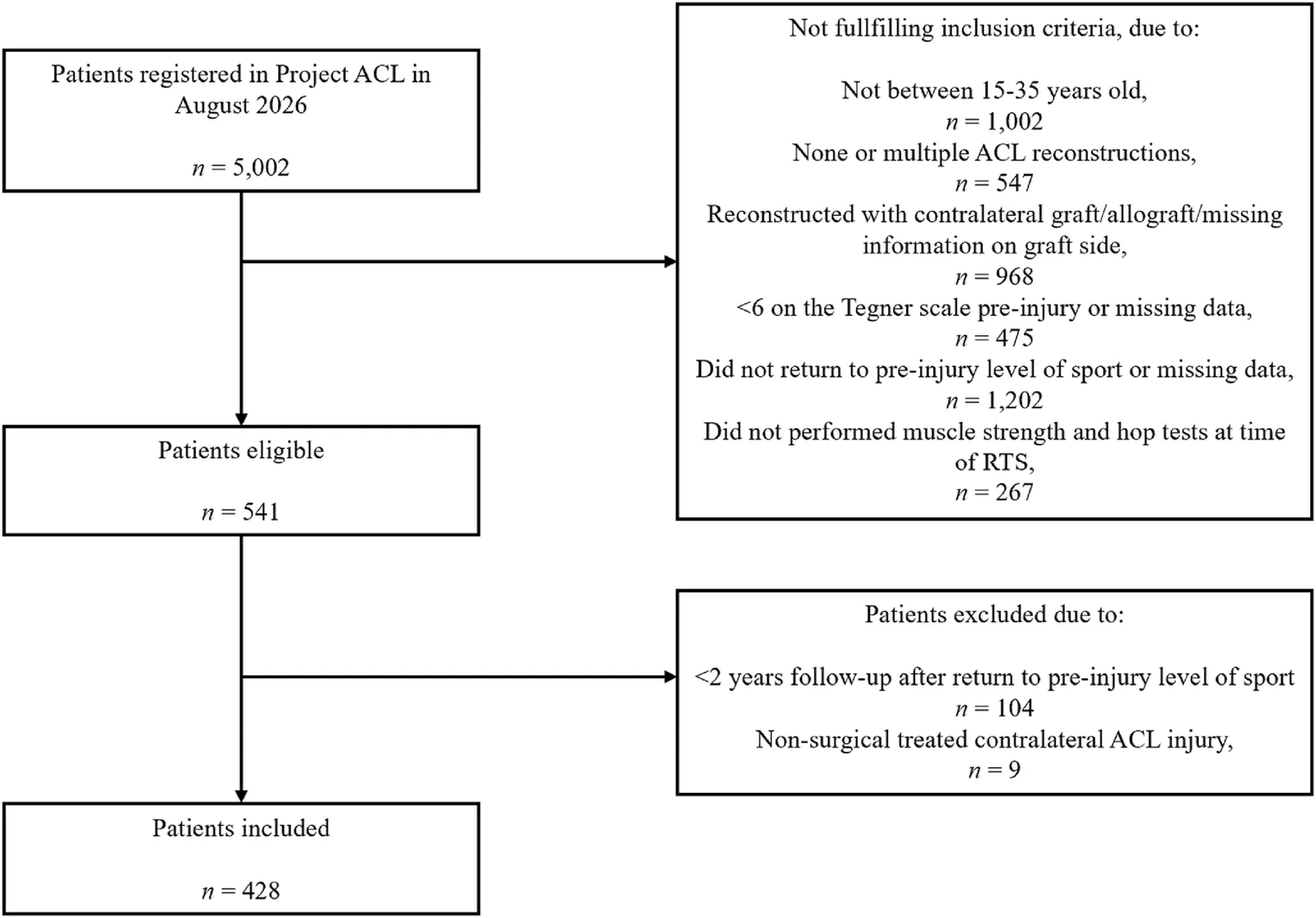
Flowchart of selection process. ACL = Anterior cruciate ligament; n = number of patients.

### 3.1 Demographics

The average age at ACLR was 21.5 ± 4.9 years, 50% of patients were female, and the average body mass index was 23.4 ± 2.6. Fifty (11.7%) patients sustained a second ACL injury within 2 years from return to pre-injury sport level, of which 31 (62%) were ipsilateral and 19 (38%) were contralateral. The median time from return to pre-injury sport level to second ACL injury was 6.6 months (25th – 75th percentiles, 3.9; 12.8 months) (Table 1). There were no differences in relative muscle strength or hop performance between patients who sustained and did not sustain a second ACL injury (Table 2).

**Table 1:** Demographic data. Mean presented with standard deviation unless other is stated.

	Total (*n* = 428)	No second ACL injury within 2 years from RTS (*n* = 378)	Second ACL injury within 2 years from RTS (*n* = 50)	*p*-value
**Sex,** female, *n* (%)	213 (49.8%)	189 (50%)	24 (48%)	0.88
**Age at ACLR,** years	21.5 (4.9)	21.6 (5.0)	20.7 (4.0)	0.15
**Body Mass index,** kg/m^2^	23.4 (2.6)	23.4 (2.6)	23.5 (2.5)	0.74
**Graft, n (%)**				0.27
*Hamstring tendon*	339 (79.2%)	296 (78.3%)	43 (86.0%)	
*Patellar tendon*	89 (20.8%)	82 (21.7%)	7 (14.0%)	
**Pre-injury Tegner level (median with 25**th**; 75**th **percentiles)**	8 (8; 9)	8 (8; 9)	8 (8; 9)	0.47
**Time to RTS from ACLR, months (median with 25**th**; 75**th **percentiles)**	11.9 (8.2; 17.8)	11.9 (8.2; 17.8)	11.9 (8.1; 17.6)	0.56
**Side of second ACL injury, n (%)**				
*Ipsilateral*			31 (62%)	
*Contralateral*			19 (38%)	
**Time to second ACL injury from RTS, months (median with 25**th**; 75**th **percentiles)**			6.6 (3.9; 12.8)	
**Time to ipsilateral second ACL injury from RTS, months (median with 25**th**; 75**th **percentiles)**			4.9 (2.2; 10.2)	
**Time to contralateral ACL injury from RTS, months (median with 25**th**; 75**th **percentiles)**			8.5 (5.7; 16.2)	

ACL = Anterior cruciate ligament; kg = kilogram; m = meters; n = number of patients; RTS = Return to pre-injury sport level.

**Table 2:** Relative strength and hop values of the ACLR and uninjured legs are reported. Mean presented with standard deviation unless other is stated.

	Total (*n* = 428)	No second ACL injury within 2 years from RTS (*n* = 378)	Second ACL injury within 2 years from RTS (*n* = 50)	*p*-value
**Relative quadriceps strength at RTS, ACLR leg (Nm/kg)**	2.73 (0.66)	2.73 (0.66)	2.76 (0.63)	0.71
**Relative quadriceps strength at RTS, uninjured leg (Nm/kg)**	2.84 (0.66)	2.85 (0.66)	2.83 (0.61)	0.83
**Relative hamstring strength at RTS, ACLR leg (Nm/kg)**	1.55 (0.44)	1.55 (0.44)	1.52 (0.41)	0.62
**Relative hamstring strength at RTS, uninjured leg (Nm/kg)**	1.57 (0.42)	1.57 (0.43)	1.55 (0.40)	0.72
**Vertical hop relative to height at RTS, ACLR leg (%)**	8.3 (2.4)	8.2 (2.4)	8.7 (2.5)	0.23
**Vertical hop relative to height at RTS, uninjured leg (%)**	9.2 (2.5)	9.1 (2.4)	9.2 (2.9)	0.81
**Hop for distance relative to body height at RTS, ACLR leg (%)**	72.1 (13.1)	71.6 (13.0)	73.6 (13.0)	0.31
**Hop for distance relative to body height at RTS, uninjured leg (%)**	76.3 (12.4)	76.2 (12.6)	77.2 (11.2)	0.58
**Total number of side hop relative to body height at RTS, ACLR leg (%)**	26.0 (7.9)	25.9 (8.0)	26.8 (7.4)	0.45
**Total number of side hop relative to body height at RTS, uninjured leg (%)**	27.1 (7.6)	27.0 (7.4)	28.2 (7.3)	0.32

ACLR = Anterior cruciate ligament reconstruction; Nm = Newton meters; n = number of patients; RTS = Return to pre-injury sport level.

### 3.2 Discriminative ability for second ACL injury

Relative muscle strength and hop performance measures demonstrated poor discriminatory ability for second ACL injury within 2 years. For any second ACL injury, AUC values ranged from 0.48 to 0.55 across the five tests. Similarly, AUC values ranged from 0.50 to 0.55 for ipsilateral second ACL injury and from 0.42 to 0.55 for contralateral ACL injury. None of the individual tests demonstrated acceptable discriminatory ability (AUC ≥0.70) for any of the three outcomes. The Youden indices were low across analyses, ranging from 0.06 to 0.14 for any second ACL injury, 0.09 to 0.18 for ipsilateral second ACL injury, and 0.07 to 0.25 for contralateral ACL injury (Table 3).

**Table 3:** Receiver operating characteristic analyses for any second ACL injury, ipsilateral second ACL injury, and contralateral ACL injury within 2 years.

	Cut-off	Youden's index	Specificity	Sensitivity	AUC
**1) Second ACL injury**					
Relative quadriceps strength, ACLR leg (Nm/kg)	2.23	0.10	0.24	0.86	0.52 (0.44; 0.60)
Relative hamstring strength, ACLR leg (Nm/kg)	1.46	0.06	0.50	0.56	0.49 (0.40; 0.57)
Vertical hop relative to body height, ACLR leg (%)	10.0	0.11	0.77	0.34	0.55 (0.46; 0.63)
Hop for distance relative to body height, ACLR leg (%)	68.7	0.10	0.42	0.68	0.54 (0.45; 0.62)
Total number of side hop relative to body height, ACLR leg (%)	25.9	0.14	0.48	0.66	0.53 (0.45; 0.61)
**2) Ipsilateral second ACL injury**					
Relative quadriceps strength, ACLR leg (Nm/kg)	2.23	0.14	0.24	0.90	0.53 (0.43; 0.63)
Relative hamstring strength, ACLR leg (Nm/kg)	1.46	0.18	0.50	0.68	0.53 (0.42; 0.63)
Vertical hop relative to body height, ACLR leg (%)	10.0	0.15	0.77	0.39	0.55 (0.44; 0.66)
Hop for distance relative to body height, ACLR leg (%)	95.5	0.10	0.97	0.13	0.52 (0.41; 0.63)
Total number of side hop relative to body height, ACLR leg (%)	25.9	0.09	0.48	0.61	0.50 (0.39; 0.61)
**3) Contralateral ACL injury**					
Relative quadriceps strength, uninjured leg (Nm/kg)	3.58	0.12	0.85	0.26	0.48 (0.33; 0.62)
Relative hamstring strength, uninjured leg (Nm/kg)	1.92	0.07	0.81	0.26	0.42 (0.27; 0.56)
Vertical hop relative to body height, uninjured leg (%)	6.1	0.10	0.10	1.00	0.44 (0.32; 0.55)
Hop for distance relative to body height, uninjured leg (%)	74.1	0.23	0.44	0.79	0.53 (0.43; 0.63)
Total number of side hop relative to body height, uninjured leg (%)	24.9	0.25	0.35	0.90	0.55 (0.45; 0.65)

ACLR = Anterior cruciate ligament reconstruction; AUC = Area under the receiver operating curve, kg = kilogram; Nm = Newton meters.

When any second ACL injury was considered, there were no significant differences in the proportion of patients meeting the calculated cut-offs between patients who sustained and did not sustain a second ACL injury. In the side-specific analyses, patients who sustained an ipsilateral second ACL injury met the calculated cut-off for the hop for distance relative to body height (*p* = 0.02) and all tests to a greater extent than patients who did not sustain an ipsilateral second ACL injury (*p* = 0.01). Patients who sustained a contralateral ACL injury met the calculated cut-offs for the total number of side hops relative to body height (*p* = 0.03) to a greater extent than patients who did not sustain a contralateral ACL injury (Appendix Table 1). Despite these differences in passing rates, the calculated cut-offs demonstrated poor overall discriminatory ability.

## 4 Discussion

Cut-off values for relative quadriceps and hamstring muscle strength and relative hop performance demonstrated poor discriminatory ability for second ACL injury within 2 years after returning to pre-injury level of sports. The result was consistent when evaluating any second ACL injury, ipsilateral second ACL injury using values from the ACLR leg, and contralateral ACL injury using values from the uninjured leg. Thus, the current tests used of muscle function interpreted as relative measures provide information about physical capacity but poorly discriminate between patients who subsequently sustain and do not sustain a second ACL injury.

### 4.1 Batteries of tests and second ACL injuries

Return to sport decision-making has been recommended to incorporate assessments of both physical function and psychological states [20]. Batteries of tests that include isokinetic strength and hop performance, interpreted as LSI, have been reported to have limited ability to identify patients at risk of sustaining a second ACL injury [4], [6]. A critique pertains to the interpretation of test results, particularly when using the uninjured leg as a reference, which may represent a ‘moving target’, as it may increase or decrease during rehabilitation [7], [8]. Relative strength and hop performance could therefore provide complementary information about physical capacity without relying on between-limb symmetry.

However, relative strength and hop performance of the ACLR leg demonstrated poor discrimination for any second ACL injury and ipsilateral second ACL injuries, while performance of the uninjured leg demonstrated poor discrimination for contralateral ACL injury. Thus, the poor discrimination was not solely explained by combining ipsilateral and contralateral injuries or by assessing only the ACLR leg. Nevertheless, patients with ipsilateral second ACL injury more frequently met the hop for distance and overall battery cut-offs, while patients with contralateral ACL injury more frequently met the side hop cut-off. Despite the differences in meeting calculated cut-offs, overall discrimination remained poor, potentially reflecting factors not captured by physical performance tests alone, including differences in sport exposure, risk appraisal or confidence, and movement quality.

The lack of clinically relevant cut-off values for discriminating between patients who sustained and did not sustain a second ACL injury does not imply that clinicians should deprioritize the recovery of strength and hop performance. Recovery of muscle function and plyometric capacity are considered important to tolerate the demands of different sports upon RTS. Rather, our findings suggest that specific cut-off values of relative muscle strength and relative hop performance are not sensitive enough or appropriate to use as an identification of patients at risk or not for second ACL injury. Thus, strength and hop performance tests remain important for assessing recovery of physical capacity and inform return to sport decision-making, however, their results should not be interpreted as stand-alone indicators of the risk of sustaining a second ACL injury [4].

### 4.2 Future directions

The poor discrimination observed raises the question whether the specific measures of quadriceps and hamstring muscle strength and hop performance assessed in the present study capture the physical characteristics relevant to second ACL injury, or whether the use of generalized cut-offs may be unrealistic. With regard to hop performance tests, the hop for distance test has been reported to mask alterations in knee function, with male athletes after ACLR demonstrating compensatory landing strategies, including less knee flexion and greater hip flexion compared with the uninjured leg [21]. A stiffer knee at landing can result in a higher strain on the ACL, with peak loading occurring at knee flexion angles between 10 and 30° [22]. Landing with a deeper knee angle reduces ACL loading despite increased quadriceps contraction [22]. Consequently, delving into the movement quality may be preferable. Butler et al. [23] reported that patients who exhibit excessive dynamic knee valgus on the involved leg during sidestep maneuvers were 4.6 times more likely to sustain a second ACL injury. Despite that the assessment movement quality during functional tests, such as sidestepping, has been reported with promising results, ACL injuries occur rapidly — within 40 ms [24] — and are often associated with an external focus, highlighting the influence of neurocognitive errors [25]. To incorporate a neurocognitive component into rehabilitation, alongside movement quality assessments, may address this gap [26]. However, further research is needed to evaluate its potential benefits in a battery of tests prior to RTS. Nonetheless, the present findings add to emerging data that performance cut-offs of a battery of tests including muscle strength and hop performance prior to return to pre-injury sport level have limited ability to discriminate between patients who sustain and do not sustain a second ACL injury [4], [5], [6].

### 4.3 Limitations

Several limitations should be considered. Sport exposure after returning to pre-injury sport level was not quantified. Although all patients had returned to their pre-injury sport level or higher according to the Tegner, the Tegner does not capture the frequency, duration, or intensity of sport participation. Additionally, isokinetic concentric muscle strength and hop performance were assessed at a single time point, while second ACL injury could occur up to 2 years later. Repeated assessments may better characterize changes in physical capacity during this period.

Normalization of muscle strength to body mass using ratio scaling (Nm/kg) assumes a proportional relationship between torque and body mass. However, our purpose was to quantify the strength available relative to the body mass that the patient is required to control during weight-bearing sporting activities, rather than solely to remove the influence of body size. Allometric scaling could provide complementary information by accounting for a potentially non-linear relationship between body mass and muscle strength and warrants investigation in future studies. Vertical hop height was estimated from flight time, which assumes comparable body configuration at take-off and landing, of which deviations from this assumptions may influence the estimated hop height [27]. Standardized instructions and testing procedures were used to minimize performance variability. Hop performance was normalized to body height as longer legs provide a greater leverage when pushing off the ground, as well as contribute to a longer stride compared to shorter legs. However, we did not have the data of leg length, only of body height as a surrogate measure of leg length. Future studies could include direct assessment of leg length to determine whether normalization to leg length provides a more appropriate anthropometric adjustment of hop performance than body height. Furthermore, greater body height may also be associated with greater mass. A greater mass may be negative for hop performance if the greater mass consists of greater fat mass [28], or positive for hop performance if the mass consists of greater fat free mass [29]. The lack of data of patients’ fat mass and fat free mass infer caution in the interpretation of our results. In addition, a greater hop for distance may, however, be a surrogate measure for patients with less kinesiophobia [30], which was not controlled for. Batteries of tests have been criticized for their failure to account for psychological factors [31], [32], while incorporation of patient-reported outcome cut-offs into physical batteries of tests has not been reported to improve their ability to identify patients at risk for second ACL injury [33].

Although 50 second ACL injuries occurred within 2 years, the side-specific analyses included only 31 ipsilateral and 19 contralateral ACL injuries. The relatively small number of events, particularly in the side-specific analyses, limits the precision of the ROC estimates and calculated cut-off values, which should therefore be interpreted cautiously.

## 5 Perspective

The findings of this study contribute to the ongoing discussion in sports medicine regarding the use of batteries of tests that consist of strength and hop performance in RTS decision-making. Previous studies [4], [6] have questioned whether LSI-based test batteries can discriminate between patients who subsequently sustain and do not sustain a second ACL injury. The present study extends this critique to cut-off values based on relative strength and hop performance. However, our findings should not diminish the importance of restoring strength and plyometric capacity, which remain critical for meeting sport-specific demands. Instead, it emphasizes the need to develop current used batteries of test to better inform decision-making prior to returning to sport. The present study reinforces the notion that commonly used test batteries are valuable for measuring recovery but should not be interpreted as stand-alone tools for identifying second ACL injury risk.

## 6 Conclusions

The calculated cut-off values for strength relative to body mass and relative hop performance were unable to differentiate between patients who sustained a second ACL injury and patients who did not within 2 years from return to pre-injury sport level. Therefore, the assessment of relative muscle strength and relative hop performance as a part of a battery of tests prior to return to pre-injury sport remain relevant measures of physical recovery after ACLR but should not be interpreted as stand-alone indicators of second ACL injury risk.

## Patient consent statement

Participation is voluntary, and informed consent is obtained during registration in the rehabilitation registry.

## Ethical approval and consent to participate

All patients received oral and written information of the project and provided informed consent. The study was approved by the Regional Ethical Review Board in Gothenburg (265–13, T023-17), and the Swedish Ethical Review Authority (2020–02501, and 2024-08724-01).

## Data availability

Data is available upon reasonable request.

## Permission to reproduce material

Not applicable.

## Authors contribution

Author JH drafted the initial version of the manuscript. Authors JH, JL, and EHS contributed substantially to the acquisition of the data and the analysis of the data, and they are responsible for drafting the manuscript and revising it critically for important intellectual content. AS, RP, RHS, RT, and KS made large contributions to the revision and design of the work. JH and EHS are responsible for the concept of design.

## Funding

None.

## Declaration of competing interest

The author declare the following financial interests/personal relationships which may be considered as potential competing interests:

KS is a board member of Getinge AB. The results of the study are presented clearly, honestly, and without fabrication, falsification, or inappropriate data manipulation.
