# Supplementary material for: Can relative muscle strength and relative hop performance cut-offs identify patients who sustain a second ACL injury?
Source: JSAMS Plus. 2026 Nov 5;8:100163. doi: 10.1016/j.jsampl.2026.100163 (PMC13753584; doi:10.1016/j.jsampl.2026.100163)
Supplement: Supplementary file 1 [file mmc1.docx]

Appendix Table 1. *Number and proportion of patients meeting the calculated cut-off values for any second ACL injury, ipsilateral second ACL injury, and contralateral ACL injury.*

|  | **Cut-off** | **No second ACL injury, *n* (%)**  **(*n* = 378)** | **Second ACL injury, *n* (%)**  **(*n* = 50)** | ***p-*value** |
| --- | --- | --- | --- | --- |
| 1) **Second ACL injury** | | | | |
| **Relative quadriceps strength, ACLR leg (Nm/kg)** | ≥ 2.23 | 289 (76.5%) | 43 (86.0%) | 0.15 |
| **Relative hamstring strength, ACLR leg (Nm/kg)** | ≥ 1.46 | 196 (51.9%) | 28 (56.0%) | 0.65 |
| **Vertical hop relative to body height, ACLR leg (%)** | ≥ 10.0 | 93 (24.6%) | 17 (34.0%) | 0.17 |
| **Hop for distance relative to body height, ACLR leg (%)** | ≥ 68.7 | 220 (58.2%) | 34 (68.0%) | 0.22 |
| **Total number of side hop relative to body height, ACLR leg (%)** | ≥ 25.9 | 198 (52.4%) | 33 (66.0%) | 0.07 |
| **All tests, n (%)** |  | 64 (16.9%) | 11 (22.0%) | 0.43 |
| 2) **Ipsilateral second ACL injury** | | | | |
|  | **Cut-off** | **No second ACL injury, *n* (%)**  **(*n* = 378)** | **Ipsilateral injury, *n* (%)**  **(*n* = 31)** | **p-value** |
| **Relative quadriceps strength, ACLR leg (Nm/kg)** | ≥ 2.23 | 289 (76.5%) | 28 (90.3%) | 0.11 |
| **Relative hamstring strength, ACLR leg (Nm/kg)** | ≥ 1.46 | 196 (51.9%) | 21 (67.7%) | 0.10 |
| **Vertical hop relative to body height, ACLR leg (%)** | ≥10.0 | 93 (24.6%) | 12 (38.7%) | 0.09 |
| **Hop for distance relative to body height, ACLR leg (%)** | ≥ 95.5 | 10 (2.6%) | 4 (12.9%) | 0.02 |
| **Total number of side hop relative to body height, ACLR leg (%)** | ≥ 25.9 | 198 (52.4%) | 19 (61.3%) | 0.36 |
| **All tests, n (%)** |  | 9 (2.4%) | 4 (12.9%) | 0.01 |
| 3) **Contralateral ACL injury** | | | | |
|  | **Cut-off** | **No second ACL injury, n (%)**  **(n = 378)** | **Contralateral injury, n (%)**  **(n = 19)** | **p-value** |
| **Relative quadriceps strength, uninjured leg (Nm/kg)** | ≥ 3.58 | 57 (15.1%) | 5 (26.3%) | 0.20 |
| **Relative hamstring strength, uninjured leg (Nm/kg)** | ≥ 1.92 | 73 (19.3%) | 5 (26.3%) | 0.55 |
| **Vertical hop relative to body height, uninjured leg (%)** | ≥6.1 | 343 (90.7%) | 19 (100.0%) | 0.40 |
| **Hop for distance relative to body height, uninjured leg (%)** | ≥ 74.1 | 211 (55.8%) | 15 (78.9%) | 0.06 |
| **Total number of side hop relative to body height, uninjured leg (%)** | ≥ 24.9 | 244 (64.6%) | 17 (89.5%) | 0.03 |
| **All tests, n (%)** |  | 39 (10.3%) | 3 (15.8%) | 0.44 |

*ACL = Anterior cruciate ligament; kg = Kilogram; Nm= Newton meters; n = number of patients.*
